# Simultaneous determination of seven hydrophilic bioactive compounds in water extract of *Polygonum multiflorum* using pressurized liquid extraction and short-end injection micellar electrokinetic chromatography

**DOI:** 10.1186/1752-153X-7-45

**Published:** 2013-03-04

**Authors:** Ka-meng Lao, Dong-qi Han, Xiao-jia Chen, Jing Zhao, Tie-jie Wang, Shao-ping Li

**Affiliations:** 1 State Key Laboratory of Quality Research in Chinese Medicine, Institute of Chinese Medical Sciences, University of Macau, Macao, China; 2 Shenzhen Institute for Drug Control, 518057, Shenzhen, P. R. China; 3 School of Pharmacy, Jiangxi Science and Technology Normal University, Nanchang, China

**Keywords:** MEKC, Polygonum multiflorum, Pressurized liquid extraction, Short-end injection

## Abstract

**Background:**

Polygoni Multiflori Radix, *He-Shou-Wu* in Chinese, is a widely used traditional Chinese medicine. Clinically, water decoction is the major application form of *He-Shou-Wu*. Therefore, simultaneous determination of bioactive compounds in water extract is very important for its quality control.

**Results:**

A pressurized liquid extraction and short-end injection micellar electrokinetic chromatography (MEKC) were first developed for simultaneous determination of seven hydrophilic bioactive compounds in water extract of *He-Shou-Wu*. The influence of parameters, such as pH, concentration of phosphate, SDS and HP-β-CD, capillary temperature and applied voltage, on the analysis were carefully investigated. Optimum separation was obtained within 14 min by using 50 mM phosphate buffer containing 90 mM SDS and 2% (m/v) HP-β-CD (pH 2.5) at 15 kV and 20°C. All calibration curves showed good linearity (*R*^*2*^>0.9978) within test ranges. The overall LOD and LOQ were lower than 2.0 μg/mL and 5.5 μg/mL, respectively. The RSDs for intra- and inter-day of seven analytes were less than 3.2% and 4.6%, and the recoveries were 97.0%-104.2%.

**Conclusion:**

The validated method was successfully applied to the analysis of *He-Shou-Wu* samples, which is helpful for its quality control.

## Background

The dried root of *Polygonum multiflorum* Thunb (*He-Shou-Wu* in Chinese) is one of the commonly used traditional Chinese medicines (TCMs) officially recorded in Chinese Pharmacopoeia. Clinically, *He-Shou-Wu* was used as a tonic and anti-aging agent in many remedies [1]. The major bioactive compounds in *He-Shou-Wu* have been reported to be stilibene and polyphenols. These compounds have multiple effects, such as antioxidation [2,3], radical scavenging activity [4], lipid regulation [5,6], hair growing effect of resting hair follicles [7], inhibition of advanced glycation end product formation [8] and neuroprotection [9-13]. Therefore, analysis of these compounds will be helpful to control the quality of *Polygonum multiflorum*. However, many analytical methods including HPLC [14-16], UPLC [17], GC [18] and CE [19,20] only focused on the analysis of anthraquinones with hepatoxic activity and stilibenes in organic solvent extract. There has been few report for determination of bioactive compounds in water extract of *He-Shou-Wu*[21,22], but LC analysis of hydrophilic compounds is still a challenge. Actually, water decoction, usually contains a lot of hydrophilic components, is the major administration form of TCMs. Therefore, analysis of hydrophilic compounds is beneficial to well understand active components in water extracts of TCMs.

CE analysis is usually performed in aqueous buffer system, which is easily used for analysis of hydrophilic components. In addition, CE also has the advantages of low consumption of reagent and sample, short analysis time and high efficiency [23,24]. Furthermore, a variety of separation modes such as CZE, MEKC, MEEKC and NACE could analyze compounds with different characteristics. To the best of our knowledge, no CE method was reported for analysis of hydrophilic bioactive compounds in *He-Shou-Wu*. This study firstly developed a pressurized liquid extraction and short-end injection MEKC method for simultaneous determination of seven hydrophilic bioactive compounds, including hypaphorine (**1**), 2,3,5,4’-tetrahydroxystilbene 2-O-β-D-glucoside (**2**), epicatechin (**3**), proanthocyanidin B2 (**4**), proanthocyanidin B1 (**5**), catechin (**6**) and gallic acid (**7**) in water extract of *He-Shou-Wu*.

### Experiment

#### Chemicals, reagents, and materials

**Figure 1:**
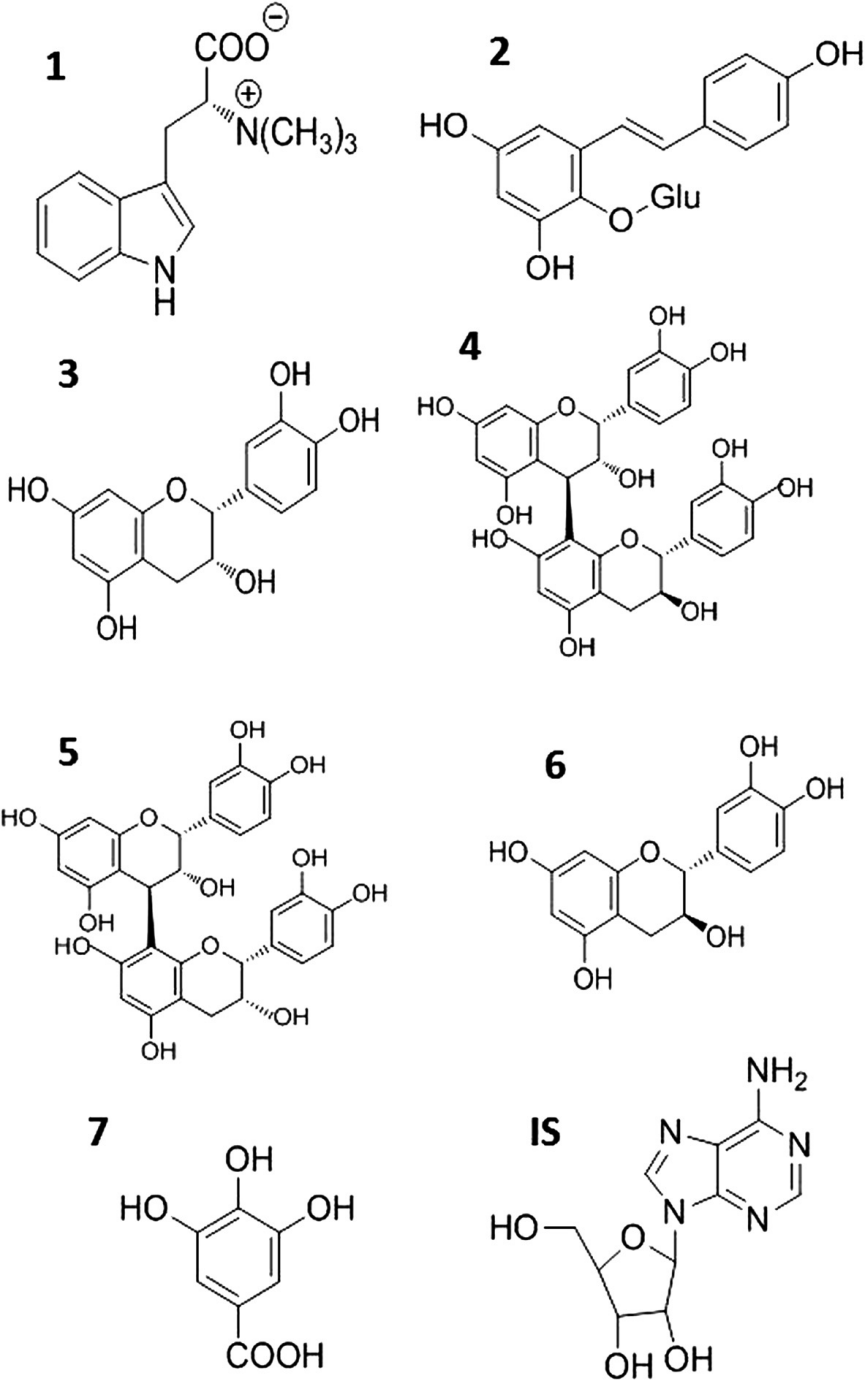
**Chemical structures of 7 investigated compounds and adenosine (internal standard, IS).** 1, hypaphorine, 2, 2,3,5,4’-tetrahydroxystilbene 2-O-β-D-glucoside, 3, epicatechin, 4, proanthocyanidin B2, 5, proanthocyanidin B1, 6, catechin, 7, gallic acid, IS, adenosine. Glu, β-D-glucose.

Catechin (>98%), epicatechin (>98%) and gallic acid (>98%) were purchased from Shanghai Winherb Medical S&T Development Co. Ltd (Shanghai, China). Proanthocyanidin B1 (>95%) and proanthocyanidin B2 (>95%) were purchased from Chengdu Biopurify Phytochemicals Co. Ltd (Chengdu, China). Adenosine was purchased from Sigma (St. Louis, MO, USA). Hypaphorine and 2,3,5,4’-tetrahydroxystilbene 2-O-β-D-glucoside (THSG) were separated and purified in our laboratory (98%, determined by HPLC). The chemical structures of the analytes and internal standard (IS) with were shown in Figure 1.

Sodium dodecyl sulfate (SDS) was purchased from USB (Cleveland, OH, USA). Sodium phosphate monobasic was purchased from Riedel-de Haën (Seelze, Germany). Hydroxypropyl-β-cyclodextrin (HP-β-CD) was purchased from DeLi Biochemical (Xian, China), poly (ethylene glycol) (PEG, Mw=1,450) was purchased from Sigma (St. Louis, MO, USA). Hydroxypropyl methylcellulose–E5 (HPMC-E5) was purchased from Colorcon (Shanghai, China). Sodium hydroxide of analytical grade was purchased from Labscan (Bangkok, Thailand). Deionized water was prepared using a Millipore Milli-Q Plus system (Millipore, Bedford, MA, USA).

The materials of *He-Shou-Wu* were collected and identified by Prof. Li Shaoping, one of the correspondence authors. The voucher specimens of these samples were deposited at the Institute of Chinese Medical Sciences, University of Macau, Macao, China.

#### Sample preparation

The extraction was performed by pressurized liquid extraction (PLE) on a Dionex ASE 200 system (Dionex, Sunnyvale, CA, USA) under the optimized conditions reported before [21]. In brief, powder (0.5 g) was mixed with diatomaceous earth in a proportion of 1:2 and placed into an 11 mL stainless steel extraction cell. The extraction cell was extracted under the optimized condition: Solvent, water; particle size, 80–96 μm; pressure, 1500 psi; temperature, 40°C; Static time, 10 min; number of cycle, 1. After PLE extraction, the extract was diluted to a certain volume in 25 mL volumetric flask with water. Before injection, the extract was filtered through a 0.45 μm filter (Millipore, Ireland) and mixed with IS in a proportion of 4:1.

Each Standard was dissolved in water as stock solution at the concentration of 1 mg/mL (10 mg/mL for THSG), and diluted to appropriate concentration, then mix with IS in a proportion of 4:1 before use.

#### MEKC analysis

All analysis was performed on an Agilent HP 3D CE instructment (Agilent Technologies, Palo Alto, CA, USA) using “Short-end injection” mode. A fused-silica capillary (64.5 cm × 75 μm id, 8.5 cm effective length; Agilent Technologies) was used throughout this study. The running buffer containing 50 mM phosphate, 90 mM SDS and 2.0% HP-β-CD was adjusted to pH 2.5 using phosphate acid. The buffer was filtered through 0.45 μm filter before it was transferred to the inlet/outlet vials. A 15 kV voltage was applied and pressure injection was 25 mbar for 3 s. The detection wavelength was 210 nm and the temperature was maintained at 20°C. The new capillary was first flushed with 1 M NaOH, 0.1 M NaOH and water for 20 min. For each run, the capillary was conditioned by rinsing with 0.1 M NaOH, water and running buffer for 3 min, respectively. Adenosine (80 μg/mL of final concentration) was used as IS.

#### Calibration curves, limit of detection and quantification

Stock solutions of reference compounds were prepared and diluted to appropriate concentrations with water, then mixed with 400 μg/mL of adenosine solution in a proportion of 4:1. At least seven concentrations of the solution were analyzed in two replicates, and the calibration curves were constructed by plotting the peak area ratio of individual standard to IS *versus* the concentration of each analyte. LOD and LOQ for each analyte were determined at an S/N of about 3 and 10, respectively.

#### Precision, repeatability and recovery

Intra- and inter-day variations were chosen to determine the precision of the developed method. For intra-day variation test, three levels of the mixed standards solution was analyzed for six replicates (n=6) within one day, while for inter-day variations test, the three levels was examined in duplicates for consecutive 3 days (n=6). Variations were expressed as RSD.

The repeatability of the method was determined by analyzing three levels (0.4 g, 0.5 g and 0.6 g) of sample HN for three replicates and represented as RSD. The recovery was performed by adding known amount of individual standards into a certain amount of sample HN. The mixture was extracted and analyzed for three replicates.

## Results and discussion

### Optimization of MEKC conditions

Due to the poor stability of several investigated components in alkaline condition [25-28], low pH was used in this study. Since the EOF and dissociation of the analytes were strongly suppressed in this acidic condition, the migration time mainly depended on the negative SDS micelles carrying the analytes to the detection windows. It would be rather long analysis time in normal injection mode (56 cm effective length) because of no EOF in such pH condition. Therefore, it is necessary to use a short-end injection mode (8.5 cm effective length) to achieve a fast separation.

**Figure 2:**
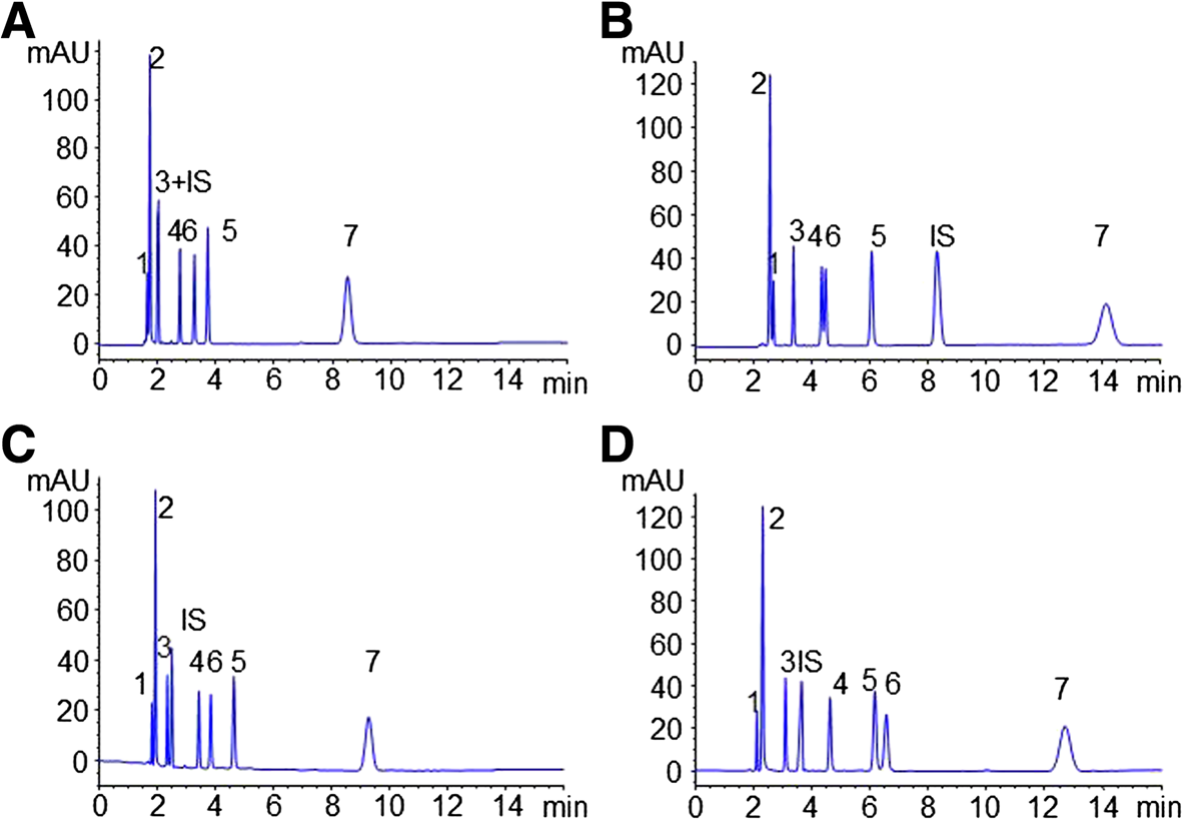
**Electrophoretograms of investigated compounds without additive (A), or with addition of 2% PEG (B), 0.5% HPMC-E5 (C) and 2% HP-β-CD (D).** MEKC Condition: Pressure injection at 25 mbar for 3 s. Running buffer containing 90 mM SDS and 50 mM phosphate (pH=2.5); voltage, 15 kV; temperature, 20°C; UV detection at 210 nm. 1–7 and IS were the same as in Figure 1.

**Figure 3:**
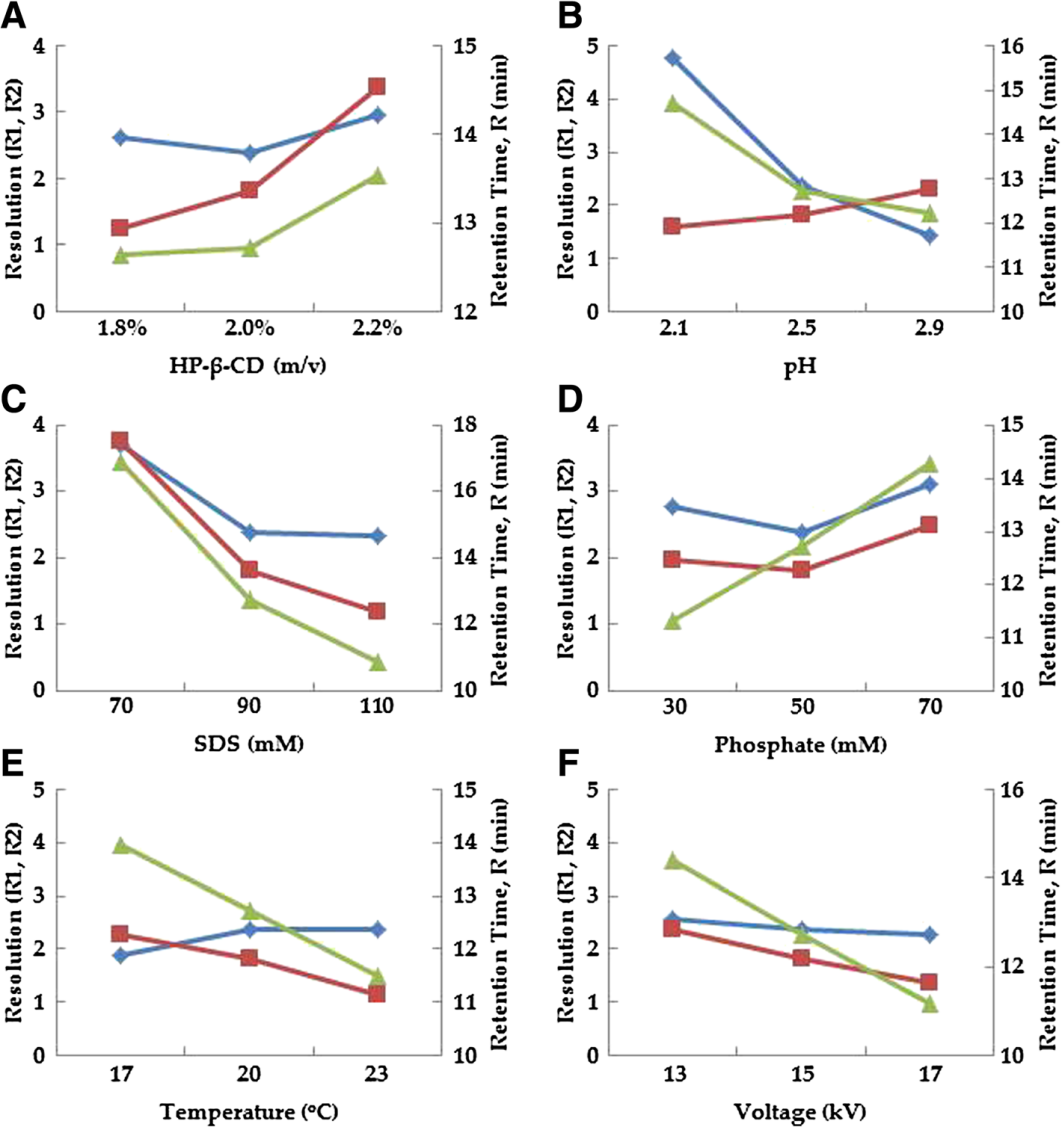
**Effect of HP-β-CD concentration (A), pH (B), SDS concentration (C), phosphate concentration (D), temperature (E) and applied voltage (F) on resolutions between hypaphorine and THSG (RHT, “blue circle symbol”), proanthocyanidin B1 and catechin (RPC, “red square symbol”), and analytical time calculated as the retention time of gallic acid (R**_**GA**_**, “green triangle symbol”).** Default MEKC condition: Pressure injection 25 mbar, 3 s. Running buffer containing 90 mM SDS, 50 mM phosphate and 2% HP-β-CD (pH=2.5); voltage, 15 kV; temperature, 20°C; UV detection at 210 nm.

Preliminary study showed that separation of analytes, especially hypaphorine and THSG, was poor (Figure 2A). It might derive from the insufficient time for separation in short-end injection mode. In order to improve the resolution, neutral additives (PEG, HPMC, HP-β-CD) were used for providing a partition effect between micelles and additives without significant change of the current. The results showed that HPMC and HP-β-CD showed obvious beneficial effect on the resolution (Figure 2). However, baseline separation between hypaphorine and THSG could not be obtained using HPMC as an additive. Especially, high viscosity of HPMC solution easily induced variation of current after several runs due to its adherence to the electrodes. Actually, HP-β-CD is a β cyclodextrin derivative which is also commonly used for enantioseparation in CE [29,30]. Finally, HP-β-CD was chosen as the neutral additive. Its concentration was also optimized based on the resolution between hypaphorine and THSG (RHT), proanthocyanidin B1 and catechin (RPC), and analytical time calculated as the retention time of gallic acid (R_GA_). Figure 3A showed that RPC improved with increase of the concentration of HP-β-CD, though analytical time was extend at high concentration of HP-β-CD. Finally, 2% HP-β-CD was added to improve separation of analytes.

As shown in Figure 3, resolution of analytes significantly decreased with increased pH though analytical time was reduced. In addition, the higher concentration of SDS, the shorter analytical time. But increased concentration of SDS induced RHT and RPC decrease. While lower concentration of phosphate buffer seems to increase both RHT and RPC, and shorten the analytical time. However, epicatechin and IS could not be separated (data not shown). Considering all mentioned above, pH 2.5, 90 mM SDS and 50 mM phosphate were selected for the analysis.

Temperature affects the buffer viscosity obviously, which leads to faster movement of micelles and then shorter analytical time. However, resolution between proanthocyanidin B1 and catechin was poor under high temperature. Similarly, RHT and RPC decreased with the increase of voltage which could induce Joule heat. Finally, 20°C and 15 kV were used for separation (Figure 3).

### Validation of the method

The linearity, LOD and LOQ of investigated analytes were determined by MEKC method under the optimum conditions. The data summarized in Table 1 indicated good relationship between the investigated compound concentrations and their peak area ratios within the test ranges (R^2^>0.9978). Their LODs and LOQs were less than 2.0 μg/mL and 5.5 μg/mL (Table 1), and intra-day and inter-day variation were less than 3.2% and 4.6%, respectively (Table 2). The repeatability (RSD, n=3) were less than 4.9%, 4.6%, and 3.3% at low (0.4 g), middle (0.5 g), and high (0.6 g) levels, respectively. The overall recovery of analytes was between 97.0%-104.2% (Table 3). The results showed the developed MEKC method was suitable for the analysis of the investigated components in water extract of *He-Shou-Wu*.

**Table 1:** Regression equation, LOQ and LOD of the analytes

**Analytes**	**Regression Equation**	**r**^**2**^	**Test range(μg/mL)**	**LOQ (μg/mL)**	**LOD (μg/mL)**
Hypohorine	y = 0.0102 x + 0.0107	0.9994	1.0-100.0	1.0	0.4
THSG	y = 0.0061 x + 0.1012	0.9988	10.9-1090.0	5.4	2.0
Epicatechin	y = 0.0241 x + 0.0327	0.9996	1.0-100.0	1.0	0.3
Proanthocyanidin B2	y = 0.0279 x + 0.0389	0.9988	1.0-100.0	1.0	0.4
Proanthocyanidin B1	y = 0.0403 x + 0.0540	0.9988	1.0-103.0	1.0	0.3
Catechin	y = 0.0365 x + 0.0592	0.9978	1.0-100.0	1.0	0.4
Gallic acid	y = 0.0662 x + 0.0929	0.9982	1.3-130.0	1.3	0.5

**Table 2:** Intra- and Inter-day variation of the investigated compounds

**Analytes**	**Concentration (μg/mL)**	**Intra-day RSD (%, n = 6)**	**Inter-day RSD (%, n = 6)**
Hypaphorine	80.0	2.2	2.0
	24.0	1.2	2.4
	8.0	3.2	4.6
THSG	872.0	1.5	1.4
	261.6	0.6	2.1
	87.2	1.8	3.1
Epicatechin	80.0	1.1	2.0
	24.0	0.6	1.8
	8.0	1.6	3.2
Proanthocyanidin B2	80.0	1.1	2.6
	24.0	1.4	1.5
	8.0	1.9	1.4
Proanthocyanidin B1	82.4	1.2	4.0
	24.7	1.6	1.5
	8.2	2.0	2.7
Catechin	80.0	0.9	3.0
	24.0	1.4	1.5
	8.0	2.4	3.4
Gallic Acid	104.0	1.1	4.4
	31.2	2.2	3.0
	10.4	2.0	4.5

**Table 3:** Recovery of the investigated analytes

**Analytes**	**Original (μg)**	**Spiked (μg)**	**Found (μg)**	**Recovery**^**a) **^**(%)**	**RSD (%)**
Hypaphorine	334.6	250.0	588.4	101.5	2.5
THSG	8215. 6	763.0	8998.3	102.6	2.5
Epicatchin	31.2	25.0	57.3	104.2	0.7
Proanthocyanidin B2	90.6	100.0	190.4	99.7	4.1
Proanthocyanidin B1	51.3	61.8	112.9	99.6	3.0
Catechin	215.9	250.0	458.4	97.0	2.2
Gallic acid	11.7	19.5	31.7	102.2	3.0

^a.)^Recovery (%) = 100 × (amount found – original amount)/amount spiked.

### MEKC analysis of seven analytes in He-Shou-Wu

**Figure 4:**
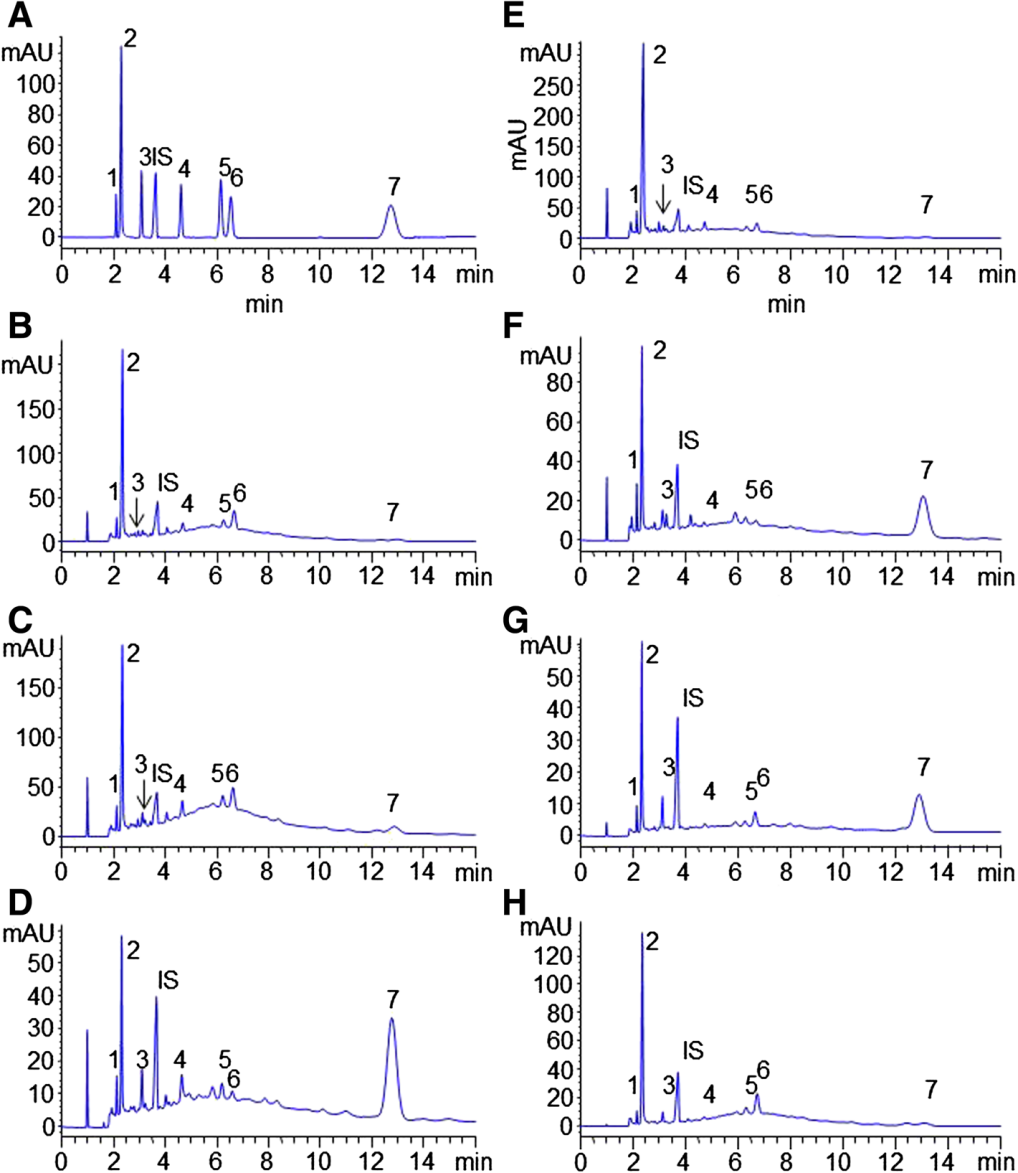
**Typical MEKC profiles of (A) mixed standards and PLE extracts of *He-Shou-Wu *from different regions (B-H).** MEKC Condition and compounds number were the same as Figure 2. Sample codes and sequence were the same as in Table 4.

The sample was prepared by optimized PLE method. The developed MEKC method was employed for the determination of seven hydrophilic compounds in different samples of *He-Shou-Wu* from different regions of China. Typical electrophoretograms were shown in Figure 4. Table 4 summarized the contents of investigated compounds in seven *He-Shou-Wu* samples. The results showed the major compound in water extract of *He-Shou-Wu* was 2,3,5,4’-tetrahydroxystilbene 2-O-β-D-glucoside, which in accordance with the previous report determined by HPLC method [21]. MEKC provided a faster separation of investigated components in *He-Shou-Wu* without consumption of organic solvent. Therefore, the developed MEKC method could be used as an alternative approach for quality control of *He-Shou-Wu*.

**Table 4:** **Contents (mg/g) of the investigated compounds in *He-Shou-Wu***

**Code**	**Sources**	**Analytes**						
		**1**^**a)**^	**2**	**3**	**4**	**5**	**6**	**7**
HN	Huaining, Anhui, China/wild	1.34 ^b)^	32.86	0.12	0.36	0.21	0.86	0.05
HS	Huangshan, Anhui, China/wild	1.50	30.40	0.40	0.80	0.48	1.28	0.55
HZ	Huzhou, Zhejiang, China/wild	0.77	7.25	0.57	0.43	0.18	0.07	3.80
QC	Qingcheng, Sichuan, China/wild	1.86	54.83	0.21	0.57	0.24	0.66	0.06
QY	Qingyang, Anhui, China/wild	1.87	15.11	0.54	0.05	0.10	0.08	2.67
SZ	Shizhu, Sichuan, China/cultivated (2 years)	0.56	7.38	0.41	+ ^c)^	+	0.22	1.36
XB	Guiyang, Guizhou, China/cultivated (2 years)	0.60	22.17	0.29	0.05	0.18	0.93	0.14

^a)^1, hypaphorine; 2, THSG; 3, epicatechin; 4, proanthocyanidin B2; 5, proanthocyanidin B1; 6, catechin; 7, gallic acid.

^b)^The data were present as average of duplicates.

^c)^Under LOQ.

## Conclusion

In this work, a fast and simple MEKC method was developed to determine seven hydrophilic bioactive compounds, including one alkaloid (hypaphorine), one stilbene (2,3,5,4’-tetrahydroxystilbene 2-O-β-D-glucoside), and five polyphenols (proanthocyanidin B1, proanthocyanidin B2, catechin, epicatechin, and gallic acid) in water extract of *He-Shou-Wu*, which is helpful to control the quality of *He-Shou-Wu.*

## Abbreviations

PLE: Pressurized liquid extraction;THSG: 2,3,5,4’-tetrahydroxystilbene 2-O-β-D-glucoside;IS: Internal standard;HPMC: Hydroxypropyl methylcellulose;PEG: Poly (ethylene glycol);HP-β-CD: Hydroxypropyl-β-cyclodextrin

## Competing interests

The authors declare that they have no competing interests.

## Authors’ contributions

SPL and JZ initiated and designed the study. The extraction and method developments were conducted by KML and DQH, and KML drafted the manuscript. All authors contributed to data analyses and to finalizing the manuscript. All authors have read and approved the final version.
